# Severity Classification of Anxiety and Depression Using Generalized Anxiety Disorder Scale and Patient Health Questionnaire: National Cross-Sectional Study Applying Classification and Regression Tree Models

**DOI:** 10.2196/72591

**Published:** 2025-09-30

**Authors:** Andre Faro, Julian Tejada, Wael Al-Delaimy

**Affiliations:** 1 Departament of Psychology, Federal University of Sergipe, Aracaju, Brazil; 2 School of Medicine, Department of Family Medicine, Herbert Wertheim School of Public Health & Human Longevity Science, University of California San Diego, 9500 Gilman Dr, La Jolla, San Diego, CA, 92093, United States, 1 8588226515

**Keywords:** anxiety, depression, machine learning, decision tree models, public mental health

## Abstract

**Background:**

Scalable and accurate screening tools are critical for public mental health strategies, especially in low- and middle-income countries (LMICs). While the Generalized Anxiety Disorder Scale (GAD-7) and Patient Health Questionnaire (PHQ-9) are widely used, their full application in large-scale programs can pose feasibility challenges. By contrast, shorter versions like GAD-2 and PHQ-2 reduce burdens but fail to capture symptom diversity.

**Objective:**

This study aimed to optimize screening for anxiety and depression severity using classification and regression tree (CART) models, identifying concise and high-performing decision rules based on the GAD-7 and PHQ-9 items, and to test their reproducibility in 5 independent datasets.

**Methods:**

A cross-sectional, nonprobabilistic study was conducted with 20,585 Brazilian adults from all 27 states and more than 3,000 cities, collected using digital outreach. Anxiety and depression symptoms were assessed using the GAD-7 and PHQ-9. CART models were trained and tested on bootstrapped samples (70% training, 30% testing), totaling 45,000 trees per scale. Each model used combinations of scale items and sociodemographic predictors. Robustness was evaluated via 10-fold cross-validation and evaluation across 3 hyperparameter configurations (*minsplit* and *minbucket*=500, 1000, 2000). Performance metrics included accuracy, sensitivity, specificity, precision, F1-score, and area under the curve (AUC).

**Results:**

The CART models produced concise, high-performing decision rules—using only 2 items for the GAD-7 and 3 for the PHQ-9. No sociodemographic variable appeared in the final classification paths. For GAD-7, the models achieved an accuracy of 86.1% for minimal or mild severity and 85.1% for severe cases, with both categories showing AUC values above 0.900. By contrast, the moderate severity class had lower performance, with accuracy around 51% and an AUC of 0.728. For PHQ-9, the models achieved 81.7% accuracy for minimal or mild cases and 78.8% for severe cases, with AUCs again exceeding 0.900 for the extreme classes; the moderate or moderately severe class showed 66.9% accuracy and an AUC of 0.776. The most frequently repeated rules included the following: “GAD2<2 and GAD4<2” for identifying minimal or mild anxiety and “GAD2≥2 and GAD4=3” for severe anxiety; for depression, “PHQ2<2and PHQ4<2” for minimal or mild cases and “PHQ2≥2 and PHQ8≥2” for severe cases. These rule-based models demonstrated stable performance across thousands of bootstrapped replications and showed reproducibility in 5 independent datasets through external validation.

**Conclusions:**

CART models enabled simplified, symptom-specific pathways for stratifying anxiety and depression severity with high precision and minimal item burden. These rule-based shortcuts offer an efficient alternative to fixed short forms (eg, GAD-2, PHQ-2) by preserving symptom diversity and severity discrimination. The findings support and lay the groundwork for adaptive, cost-effective screening and intervention models, especially in resource-limited settings and LMICs.

## Introduction

Public mental health has become a central focus in the 21st century due to the global mental illness epidemic [1,2]. The extensive global impact of the COVID-19 pandemic has increased the urgency for public mental health [3,4]. Common mental disorders (CMDs), particularly anxiety and depression, are significant challenges for public health institutions in both high-income and low- and middle-income countries (LMICs) for the foreseeable future, with the latter bearing a disproportionately higher burden [5-9]. Even before the pandemic, high CMD prevalence underscored the need for tailored national and global strategies [1,10-12].

At national levels, comprehensive mental health strategies must include regular screening and a care model capable of addressing the full spectrum of needs—from prevention and basic psychological care to outpatient interventions and emergency support during crises [1,13]. Such an approach should be routine, not limited to critical periods; include health professional training to provide basic mental care, equipping specialists and nonspecialists to address anxiety and depression symptoms (at least regarding basic psychological care); and ensure resource availability during high-pressure periods [2,14]. A robust mental health surveillance system capable of detecting varying CMD risk levels can play a vital role in building an effective care system [6,8,15]. Using brief protocols or simple instruments to stratify risks can provide actionable insights and support targeted intervention planning [16-19].

A care model with tailored strategies based on the presence and severity of health outcomes is more feasible if it is brief; moreover, reliable tools are available for both individual assessments and widespread screenings [20-24]. The Generalized Anxiety Disorder Scale-7 (GAD-7) and Patient Health Questionnaire-9 (PHQ-9) are widely used for measuring anxiety and depression symptoms, respectively, and match several important qualities [25-27]. However, they may pose a burden on participants in large-scale mental health initiatives, especially in resource-limited settings, in which the GAD-2 and PHQ-2 are used as alternatives. Notwithstanding, these shorter versions do not differentiate mental health condition severity and revert to the first 2 questions after a binary screening diagnostic [28-30], rather than more specifically screening the severity or specific symptoms of anxiety or depression.

By focusing on only two symptoms of each disorder—nervousness and worrying in GAD-2 and anhedonia and hopelessness in PHQ-2—these scales overlook other relevant symptoms, such as fatigue, sleep disturbances, difficulty concentrating, and irritability (common in anxiety disorders) or changes in appetite, guilt, and suicidal ideation (common in depression) [31]. Consequently, they are limited in capturing important symptom profiles across diverse populations, which is crucial for tailoring interventions. This restricts a comprehensive understanding of anxiety and depression when compared to the broader symptomatology outlined in the Diagnostic and Statistical Manual of Mental Disorders (DSM-5) [31].

Machine learning (ML), a subfield of artificial intelligence (AI), identifies hidden patterns in datasets through data-driven algorithms, enabling explanatory or predictive model creation [32-35]. In the mental health field, ML models provide evidence of predicting risks and streamlining assessments [36-40]. ML algorithms can support shorter, more accurate tool development, addressing challenges such as participant fatigue and low response rates while maintaining scalability and cost-effectiveness as end points [41,42]. Furthermore, within ML algorithms, decision tree models offer a distinct approach to streamlining the path from individual items to diagnostic classification (eg, by identifying the most relevant features, thereby preserving predictive accuracy and improving efficiency). By incorporating additional variables (eg, sociodemographic data, psychological constructs, and other health outcomes), decision trees allow for adaptive and personalized analyses [43-46]. ML-based scale reduction techniques, combined with logical “shortcuts” (or rules) for case classification, offer a promising approach to balancing precision and brevity in public mental health.

This study aims to leverage ML algorithms, particularly decision tree models, to enhance the efficiency and precision of GAD-7 and PHQ-9 assessments by identifying the most predictive components across severity levels. To further examine the robustness and generalizability of the derived rules, their performance was evaluated across 5 independent datasets, including 1 collected nationwide in Brazil and 4 from international samples. We hypothesized that classification and regression tree (CART) models can identify concise, high-performing decision rules using less items from the GAD-7 and PHQ-9 scales, enabling accurate stratification of disorders into minimal/mild, moderate, and severe categories, thereby optimizing mental health screening in public health applications.

## Methods

### Study Design

This study adopted a cross-sectional, nonprobabilistic design and used a digital convenience sampling method. Data were collected between March and June 2024 through online recruitment via social media, primarily Instagram and Facebook. Digitally boosted posts promoted participation across all regions of Brazil. A university-based research laboratory specializing in public mental health managed the campaign through its official page. Upon accessing the study link, participants were directed to a landing page describing the study objectives and procedures. Participants completed the survey independently using their own devices in home or personal settings, without any direct researcher supervision. Only individuals aged 18 years or older could participate. The final sample included 20,585 respondents from all 27 Brazilian states and more than 3,000 cities, encompassing both rural and urban regions. The average completion time for the questionnaire was approximately 5 minutes. Incomplete responses were excluded, resulting in a withdrawal rate of approximately 8%.

### Ethical Considerations

This study was approved by Brazil’s National Council for Ethics in Research (Conselho Nacional de Ética em Pesquisa [CONEP]; protocol number 30485420.6.0000.0008). All procedures complied with Brazilian and international regulations for research involving human participants. Informed consent was obtained electronically from all participants before survey participation. Participants were first presented with the study objectives, procedures, and data protection measures. Only individuals who explicitly agreed to participate were then granted access to the survey instruments. All data were collected anonymously, and no personally identifiable information was recorded. The dataset was anonymized before analysis to ensure confidentiality and data protection, and no supplementary materials contain any information that could lead to the identification of individual participants. In accordance with Brazilian research ethics policies involving human subjects, no monetary or material compensation was provided to participants. No generative AI tools were used in the manuscript writing, ideation, or drafting.

### Instruments

The GAD-7 is a 7-item measure for assessing anxiety [47], with each item rated on a scale from 0 (not at all) to 3 (nearly every day) and the final score ranging from 0 to 21. It has demonstrated satisfactory psychometric properties and validity evidence in Brazil [48]. Specifically, studies using the Brazilian-Portuguese version have reported adequate internal consistency (eg, Cronbach α values typically above .80) and construct validity, supporting its use for assessing generalized anxiety symptoms in this population. The PHQ-9 is a 9-item scale for assessing depression [49] with the same Likert response format as the GAD-7 and the total score ranging from 0 to 27. It has also shown satisfactory psychometrics in the Brazilian population [50] with good internal consistency (eg, Cronbach α often exceeding .85) and strong evidence of both construct and criterion validity, making it a reliable tool for depression assessment [51]. Additionally, a sociodemographic questionnaire was administered, including items on sex/gender, age, education level, and self-reported skin color/ethnicity.

A common cutoff score of 10 is often used for GAD-7 and PHQ-9 to distinguish clinical from nonclinical cases. However, alternative stratifications based on severity levels offer a more nuanced differentiation between subclinical and extreme cases (see [27]). One alternative involves classifying noncases (“minimal or mild” severity, scores <10 on both GAD-7 and PHQ-9) and cases, including the stratification of 2 levels of severity: “moderate” (“moderate” [GAD-7, scores 10‐14] and “moderate and moderately severe” [PHQ-9, scores 10‐19]) and “severe” (GAD-7, score ≥ 15 and PHQ-9, score ≥ 20). We adopted this tripartite stratification due to its parsimonious approach to prioritizing individuals as it supports different care strategies under a straightforward interpretation.

### Decision Tree

The CART method, originally proposed by Breiman, Friedman, Olshen, and Stone [52], is one of the most widely used recursive partitioning decision tree algorithms and implemented through the rpart package in R. This ML algorithm begins with the complete dataset and recursively partitions it into smaller subsets based on both the predictor values and the outcome classes (see [53-56]). In this study, the classes were defined by the trichotomized GAD-7 and PHQ-9 scores, whereas the variables included both the scale items and sociodemographic factors.

### Data Modeling

**Figure 1.**
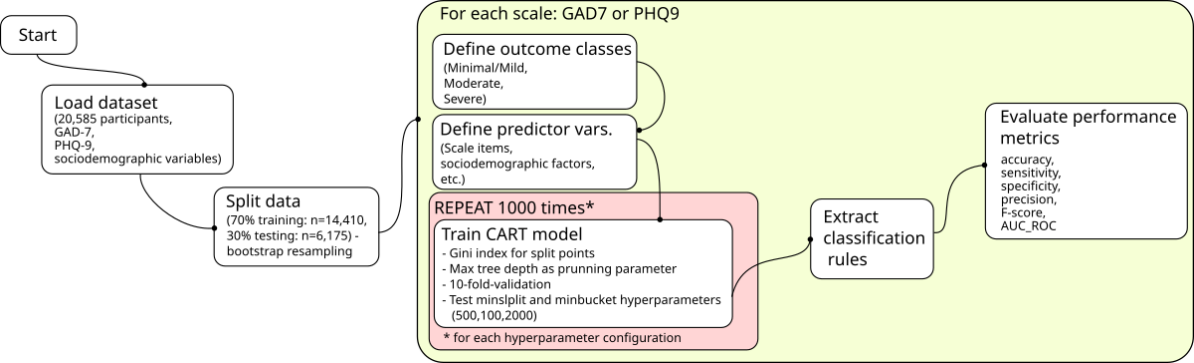
Flowchart of the data modeling procedure for generating decision tree rules to classify anxiety and depression severity in a national sample of Brazilian adults (2024). CART: classification and regression tree; GAD-7: Generalized Anxiety Disorder Scale; PHQ-9: Patient Health Questionnaire (PHQ-9).

A combination of bootstrapping and cross-validation was used to mitigate overly optimistic estimations. In this, the entire dataset was resampled multiple times, with 70% of the samples (n=14,410) used for training the ML model and the remaining 30% (n=6175) for testing its performance. This approach helps incorporate sample variability while reducing the risk of overfitting by ensuring the model is not trained on a single dataset. Generalization performance was assessed by estimating accuracy and area under the receiver operating characteristic curves (AUC-ROC) for each repetition. Classification trees were constructed using the Gini index method to determine split points, with maximum tree depth as a pruning parameter and a 10-fold cross-validation strategy implemented via the rpart2 command from the caret package. To test model robustness, the following values for the hyperparameters minsplit (minimum number of observations required in a node) and minbucket (minimum number of observations in a terminal node) were systematically explored: 500, 1000, and 2000. These values were chosen to represent a range from more flexible (500, allowing for smaller nodes and potentially more complex trees) to more constrained (2000, leading to larger nodes and simpler trees), ensuring that the identified rules were not overly specific to a single set of parameters and thus enhancing generalizability (see Figure 1). Each hyperparameter configuration was tested 1000 times to assess the stability and consistency of the derived rules across multiple bootstrapped samples (see [57-59]).

Each scale was analyzed separately, with GAD-7 and PHQ-9 scores as predictor variables (features) and severity levels from each scale as outcomes (targets). Four additional models for each scale incorporated demographic variables as additional features. From the 45,000 decision trees generated for each scale, classification rules for each class were extracted. The most frequent rules for each class were evaluated using common performance metrics, including accuracy, sensitivity, specificity, precision, F-score, and AUC-ROC curve. These metrics are widely recognized for classifier performance evaluation (see [60]).

The confusion matrix compared actual classifications (cutoffs) with model predictions. For example, GAD-7 scores categorized anxiety as minimum/mild (0‐9), moderate (10-14), or severe (15+). Correct classification of a severe case (eg, score 16) is a true positive, and misclassification is a false negative. Similarly, if a nonsevere case (eg, score 6) is correctly classified, it is a true negative and otherwise, a false positive. Confusion matrices summarize classification accuracy, with higher accuracy reflecting better alignment between predicted and actual severity levels. Sensitivity measures the model’s ability to detect true positives, while specificity assesses correct identification of true negatives. Higher sensitivity ensures better detection of severe cases, and higher specificity improves nonsevere classification. Precision minimizes false positives, with the F-score balancing precision and recall. The AUC-ROC curve evaluates performance by plotting the true-positive versus false-positive rates, with higher values indicating better classification (see [60]). Variable importance analysis examined demographic predictors’ impact on model performance.

### External Validation

To evaluate the stability and generalizability of the rules derived from the main sample, we performed an external validation with 5 datasets: 4 collected by other research groups and available in open repositories and 1 collected between November and December 2024 in Brazil using the same recruitment procedures as in the primary study. A description of these datasets is provided in Table 1 of the Supplementary Material. The rules identified in the main analyses were applied unchanged to these datasets to assess reproducibility and to verify that the models were not overfitted to the initial sample but could generalize to a broader population. Model performance was assessed using the same metrics used in the main analyses: accuracy, sensitivity, specificity, precision, and F1-score for each class of each rule.

### Software

R (R Development Core Team, 2024) plus RStudio were used to perform all analyses. The decision trees were built using the combination of the *rpart* and *caret* R packages [61] to perform cross-validation as a pruning criterion. Additionally, the values of 500, 1000, and 2000 and their combinations were used to set the *minsplit* and *minbucket* hyperparameters. The R package *pROC* [62] was used to estimate the AUC-ROC curve. All R scripts used for the data analysis are available at the Open Science Framework (OSF) repository [63].

## Results

### Overview

Based on the total sample, more than 90% were women (n=18,844, 91.5%). Approximately half of the participants declared having white skin color (n=10,405, 50.5%) and high school level education (n=10,083, 49%). The most common age groups were 40‐49 (n=6071, 29.5%) and 30‐39 years (n=5927, 28.8%). The mean age was 41.1 years (SD=12.9, range=18‐80) (Table 1).

**Table 1.** Sociodemographic profile of participants in a cross-sectional study on anxiety and depression symptoms in Brazilian adults (n=20,585), 2024.

Variables	Main sample (n=20,585)
Sex/gender, n (%)	
Male	7.6 (1564)
Female	91.5 (18,844)
Nonbinary	0.9 (177)
Skin color/ethnicity, n (%)	
White	50.5 (10,405)
Black	10.5 (2153)
Parda (mixed race)	36.7 (7575)
Other	2.2 (452)
Education level, n (%)	
Up to high school	36.3 (7062)
Undergraduate students	16.7 (3440)
Graduate school	49.0 (10,083)
Age group (years), n (%)	
18‐29	18.1 (3730)
30‐39	28.8 (5927)
40‐49	29.5 (6071)
50‐60	14.9 (3062)
More than 60	8.7 (1795)

### Generalized Anxiety Disorder Scale-7

**Figure 2.**
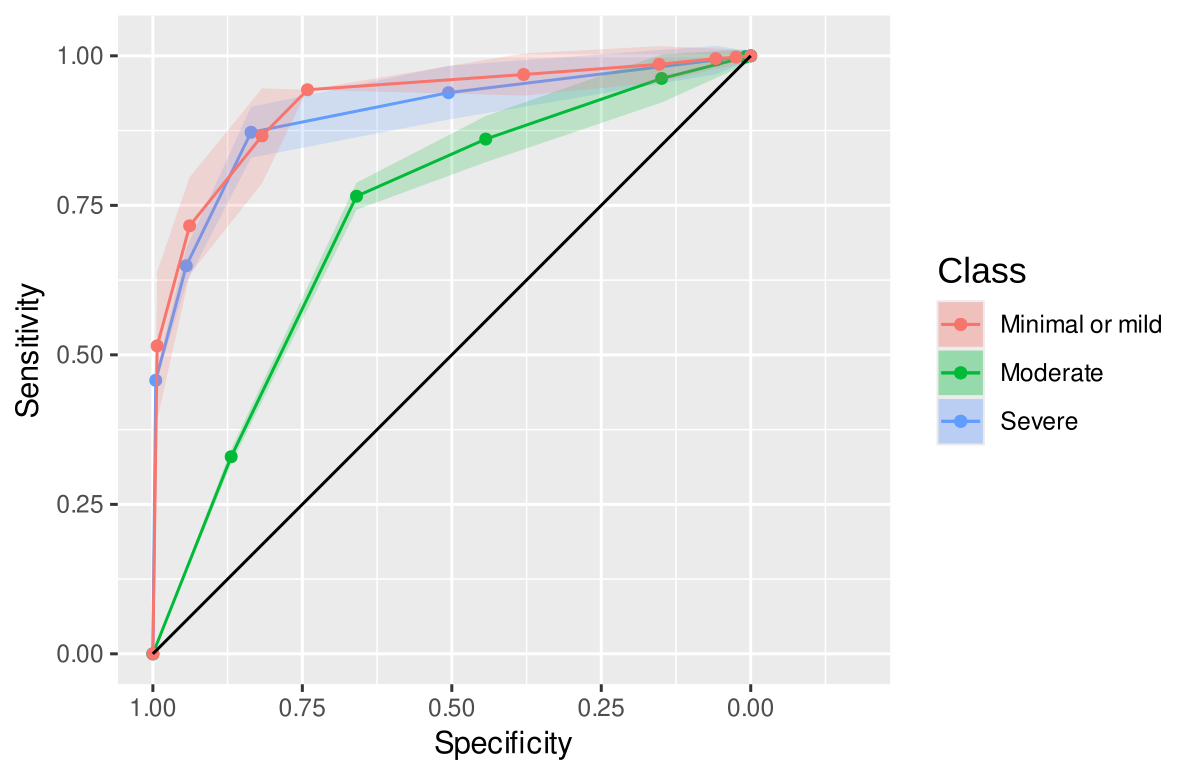
ROC curves for the most frequent decision tree rules classifying GAD-7 severity levels in a cross-sectional study of Brazilian adults (n=20,585), 2024. Each ROC curve represents the classification performance of the most frequently repeated decision tree rule for a given GAD-7 severity level (minimal, mild, moderate, or severe). The colored ribbons around the curves indicate the SD across 45,000 bootstrapped replications. GAD-7: Generalized Anxiety Disorder scale; ROC: receiver operating characteristic.

All trees for the GAD-7 scale showed satisfactory accuracy with a mean of 0.742 and over 0.850 for the “minimal or mild” and “severe” levels, respectively. In total, 159 distinct rules were identified, collectively repeated 206,900 times across the trees, and no demographic variable appeared as part of any rule. The most repeated rules from each GAD-7 class (see Table 2) accounted for more than 59% of all rule repetitions. Table 2 presents the rules for each GAD-7 severity class along with the mean and SD of several classification performance metrics. The most frequently repeated rules for the “minimal or mild” and “severe” severity levels exhibited the highest performance across the metrics. The “moderate” level demonstrated a lower performance than others but with satisfactory indices. Figure 2 displays the ROC curves estimated for all repetitions of the most frequently repeated rules.

**Table 2.** Performance metrics for the most frequently repeated decision tree rules for each severity level of the Generalized Anxiety Disorder (GAD) Scale-7 in a cross-sectional study of Brazilian adults (n=20,585), 2024.^a^

Class	Rule	Repetition	Accuracy	Sensitivity	Specificity	Precision	F-score	AUC^b^
Minimal or mild	GAD2<2 and GAD4<2	43155	0.861(0.003)	0.744 (0.023)	0.942 (0.009)	0.857 (0.015)	0.796 (0.008)	0.923 (0.004)
Moderate	GAD2<2 and GAD4≥2	41780	0.515 (0.066)	0.606 (0.054)	0.798 (0.031)	0.534 (0.021)	0.566 (0.019)	0.728 (0.013)
	GAD2≥2 and GAD4<3	9270	0.507 (0.005)	0.680 (0.009)	0.749 (0.005)	0.507 (0.008)	0.580 (0.007)	0.728 (0.013)
Severe	GAD2≥2 and GAD4=3	27800	0.851 (0.033)	0.831 (0.063)	0.877 (0.030)	0.824 (0.025)	0.825 (0.022)	0.902 (0.009)

a Values for accuracy, sensitivity, specificity, precision, F1-score, and AUC are reported as mean (SD) across 45,000 bootstrapped decision tree models.

b AUC: area under the curve.

**Figure 3.**
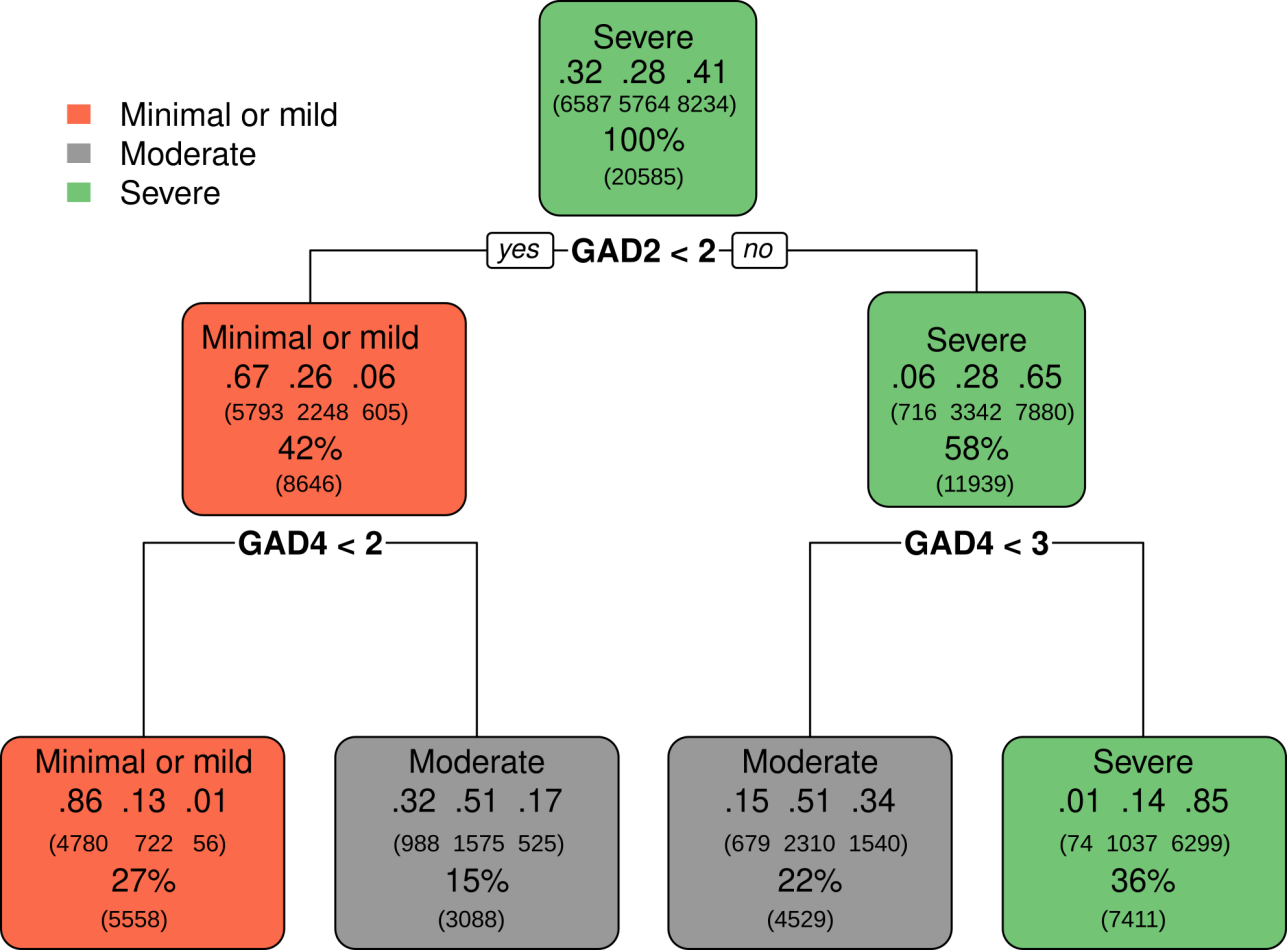
Prototypical decision trees representing the most frequently repeated classification rules for each severity level of the GAD-7 scale in a cross-sectional study of Brazilian adults (n=20,585), 2024. Each node displays the classification output of a decision rule for a specific GAD-7 severity level (minimal or mild, moderate, and severe). The middle line of each rectangle shows decimal values representing the proportion of participants in each true severity class, ordered left to right: minimal or mild, moderate, and severe. These values indicate the actual distribution of GAD-7 scores among those classified by the rule. The percentage on the bottom line indicates the proportion of the total sample that followed the corresponding decision path. GAD-7: Generalized Anxiety Disorder scale.

The most frequently repeated schematic rules for each GAD-7 class as prototypical decision trees are illustrated in Figure 3. These include the “minimal or mild” level and are characterized by the rule “GAD2<2 and GAD4<2.” This means that a person who scored 0 or 1 in GAD2 (“not being able to stop or control worrying”) and GAD4 (“trouble relaxing”) was correctly classified as having “minimal or mild” level anxiety in 86% (0.86) of the cases, within a total of 27% of cases that matched with this class. Of the 27% classified as “minimal or mild,” 13% were misclassified as “moderate” (0.13) and almost none as “severe” (0.01).

The rules were similar for cases from moderate severity levels or positive cases at any level up to significant anxiety symptoms. For example, to classify the “severe” level, the rule was “GAD2≥2 and GAD4=3.” Of the participants scoring more than or equal to 2 in GAD2, 58% were included in the “severe” level, with 65% (.65) correctly classified. By adding GAD4 equal to 3, the percentage of cases classified as “severe” reduced to 36%, but the proportion of correctly classified cases increased to 85% (.85), with almost no cases misclassified as “minimal or mild” (.01). This means that the additional item reduced false-positive cases while increasing the accuracy level. The “moderate” levels showed 2 rules and 2 paths, implying more complexity: (a) “GAD2<2 and GAD4≥2,” with 15% of total cases, of which 51% (.51) were correctly classified and (b) “GAD2≥2 and GAD4<3” with 22% of the total cases, of which 51% (.51) were correctly classified through the other path.

Beyond statistical performance, the clinical interpretation of these decision rules offers valuable insights. For the “minimal or mild” anxiety level, the rule “GAD2<2 and GAD4<2” suggests that individuals who report “not being able to stop or control worrying” (GAD2) and “trouble relaxing” (GAD4) “not at all” or “several days” (scores of 0 or 1) are highly likely to be correctly classified as having minimal or mild anxiety. By contrast, for the “severe” anxiety level, the rule “GAD2 ≥2 and GAD4=3” indicates that individuals who report “not being able to stop or control worrying” “more than half the days” or “nearly every day” (GAD2 score ≥2) and “trouble relaxing” “nearly every day” (GAD4 score=3) are highly indicative of severe anxiety. This specific combination underscores a significant and pervasive functional impairment related to anxiety symptoms, where both cognitive (worry) and somatic/behavioral (relaxation) aspects are severely affected. The addition of GAD4=3 to the rule for severe cases importantly reduces false positives, indicating that a high level of trouble relaxing is a critical differentiating factor for severe anxiety.

### Patient Health Questionnaire-9

**Figure 4.**
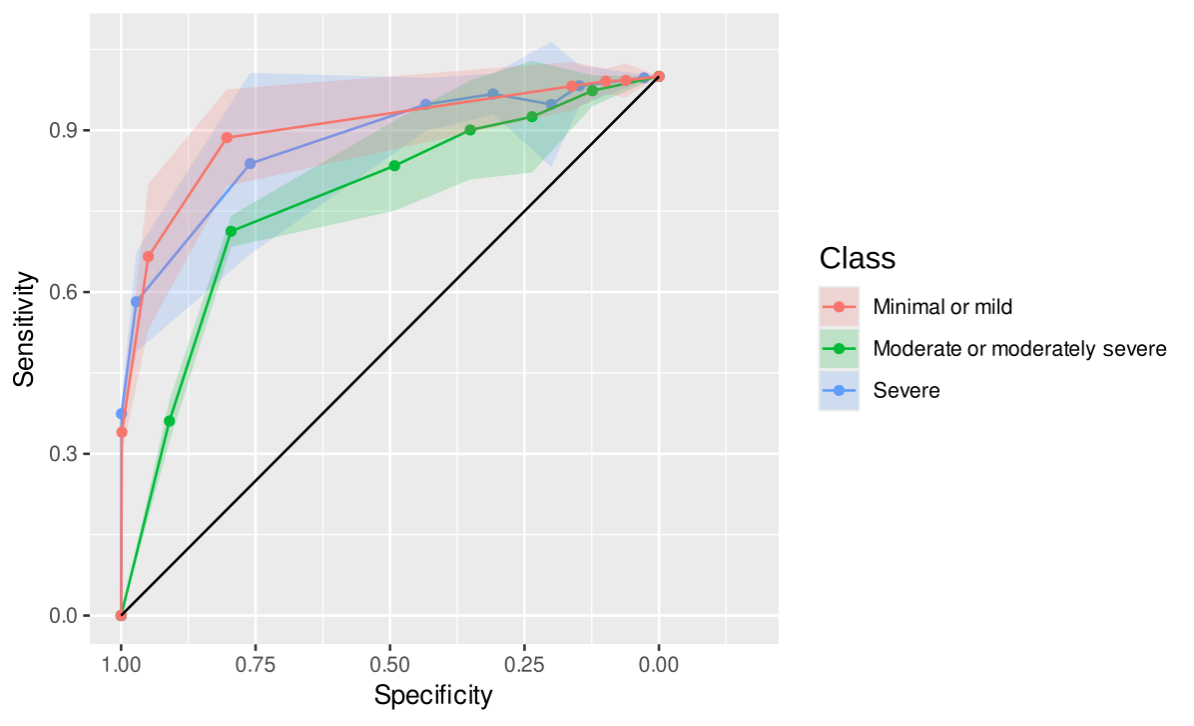
ROC curves for the most frequent decision tree rules classifying PHQ-9 severity levels in a cross-sectional study of Brazilian adults (n=20,585), 2024. Each ROC curve represents the classification performance of the most frequently repeated decision tree rule for a given PHQ-9 severity level (minimal, mild, moderate, moderately severe, or severe). The colored ribbons around the curves indicate the SD across 45,000 bootstrapped replications. PHQ-9: Patient Health Questionnaire-9; ROC: receiver operating characteristic.

The decision trees for the PHQ-9 scores also demonstrated satisfactory accuracy, with a mean of 0.757 and “minimal or mild” and “severe” levels close to 0.800. In total, 126 distinct rules were identified and repeated 230,630 times across the trees. Similar to the GAD-7, no demographic variable appeared as part of any rule. The most repeated rules for each PHQ-9 class (Table 3) accounted for more than 51% of all rule repetitions. Table 3 presents such rules for each PHQ-9 severity class and the classification performance metrics. The “minimal or mild” and “severe” severity levels of the PHQ-9 also exhibited the highest performance across the estimated metrics. The fit was lower in the “moderate level,” although with acceptable indicators. Figure 4 displays the ROC curves for all rule repetitions.

**Table 3.** Performance metrics for the most frequently repeated decision tree rules for each severity level of the PHQ-9 scale in a cross-sectional study of Brazilian adults (n=20,585), 2024.^a^

Class	Rule	Repetition	Accuracy	Sensitivity	Specificity	Precision	F1-score	AUC
Minimal or mild	PHQ2<2 and PHQ4<2	28740	0.817 (0.004)	0.767 (0.008)	0.930 (0.003)	0.817 (0.007)	0.792 (0.006)	0.921 (0.011)
Moderate or moderately severe	PHQ2≥2 and PHQ8<2	32125	0.669 (0.004)	0.783 (0.041)	0.723 (0.023)	0.677 (0.012)	0.725 (0.016)	0.776 (0.029)
	PHQ2<2 and PHQ4≥2	29680	0.688 (0.005)	0.761 (0.039)	0.733 (0.032)	0.679 (0.019)	0.717 (0.011)	0.776 (0.029)
Severe	PHQ2≥2 and PHQ8≥2	26405	0.788 (0.003)	0.700 (0.041)	0.921 (0.010)	0.782 (0.012)	0.738 (0.017)	0.903 (0.011)

a Values for accuracy, sensitivity, specificity, precision, F1-score, and AUC are reported as mean (SD) across 45,000 bootstrapped decision tree models. Precision, F1-score, and AUC are reported as mean (SD) across 45,000 bootstrapped decision tree models.

b AUC: area under the curve.

**Figure 5.**
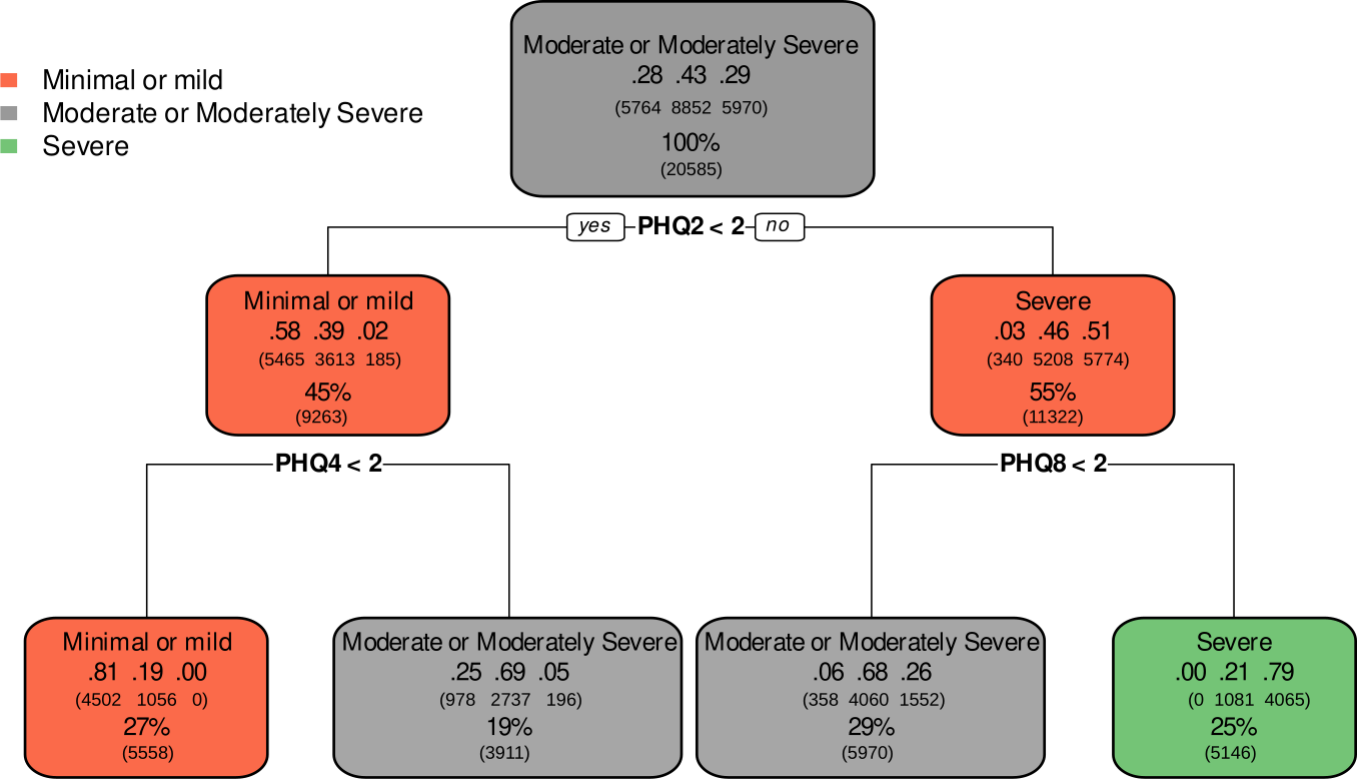
Prototypical decision trees representing the most frequently repeated classification rules for each severity level of the PHQ-9 scale in a cross-sectional study of Brazilian adults (n=20,585), 2024. Each node shows the classification output of a decision rule for a specific PHQ-9 severity level (minimal or mild, moderate or moderately severe, and severe). The decimal values on the middle line of each rectangle represent the proportion of participants in each true severity class, ordered from left to right: minimal or mild, moderate or moderately severe, and severe. These values indicate the actual distribution of PHQ-9 scores among participants classified by the rule. The percentage on the bottom line indicates the proportion of the total sample that followed the corresponding decision path. PHQ-9=Patient Health Questionnaire-9.

The highest repeated rules for each PHQ-9 class are illustrated as prototypical decision trees in Figure 5. The “minimal or mild” level was characterized by the rule “PHQ2<2 and PHQ4<2,” which means that a person who scored 0 or 1 on PHQ2 (“feeling down, depressed, or hopeless”) and PHQ4 (“feeling tired or having little energy”) was correctly classified as having a “minimal or mild” level depression in 81% (.81) of the 27% total cases that fit within this class. Concerning misclassifications, 10% were misclassified as “moderate or moderately severe” (.10) and none as “severe” (.0).

Considering only the “moderate” level, which can imply positive cases at some level to significant depression symptoms, the rules were based on a few items. For instance, to classify the “severe” level, the rule was “PHQ2≥2 and PHQ8≥2.” Considering the response higher or equal to 2 in PHQ2, 55% of the cases were at the severe level, with 51% (0.51) correctly classified. When the PHQ8 item (“moving or speaking so slowly that other people could have noticed. Or the opposite—being so fidgety or restless that you have been moving around a lot more than usual”) greater than or equal to 2 was added, the proportion of cases classified as “severe” decreased to 25%; however, the proportion of correctly classified cases increased to 79% (0.79), with none as “minimal or mild” (0.0). The “moderate or moderately severe” level showed 2 rules, indicating a more complex classification: (a) “PHQ2≥2 and PHQ8<2” with 29% of total cases and shared some fit with the “minimal or mild” (0.06) and “severe” levels (0.26), although 68% (0.68) of them were correctly classified and (b) “PHQ2<2 and PHQ4≥2,” with 19% of total cases, of which 69% (0.69) were correctly classified in the other route, which shared some fit with “minimal or mild” and “severe” cases (0.25 and 0.05, respectively). Finally, item 9, which addresses suicidal ideation, did not align with the final rules for the PHQ9 scale.

The clinical interpretation of the PHQ-9 decision paths reveals key depressive symptom profiles. For the “minimal or mild” depression level, the rule “PHQ2<2 and PHQ4<2” suggests that individuals reporting “feeling down, depressed, or hopeless” (PHQ2) and “feeling tired or having little energy” (PHQ4) “not at all” or “several days” (scores of 0 or 1, respectively) were accurately classified as having minimal or mild depression. This highlights that the absence of frequent anhedonia/hopelessness and fatigue is a strong indicator of lower severity. For the “severe” level, the rule “PHQ2≥2 and PHQ8≥2” was particularly revealing. It indicated that individuals experiencing “feeling down, depressed, or hopeless” “more than half the days” or “nearly every day” (PHQ2≥2) and significant psychomotor agitation or retardation (“moving or speaking so slowly.or being so fidgety or restless.” [PHQ8≥2]) are highly indicative of severe depression. The inclusion of PHQ8 points to a clinically significant functional impairment often associated with severe depressive episodes, with observable changes in motor activity.

### External Validation

When applied to the independent datasets, the rules identified in the main analyses showed consistent performance across both instruments. For the GAD-7, the rules showed high accuracy (range=0.852‐0.902, M=0.875, SD=0.025), sensitivity (0.792‐0.903, M=0.847, SD=0.047), specificity (0.916‐0.951, M=0.937, SD=0.015), precision (0.925‐0.983, M=0.961, SD=0.033), and F1-score (0.853‐0.942, M=0.900, SD=0.036) for the minimal anxiety class. For the severe class, the results were also satisfactory, with accuracy (0.865‐0.969, M=0.919, SD=0.055), sensitivity (0.585‐0.797, M=0.722, SD=0.094), specificity (0.929‐0.986, M=0.965, SD=0.025), precision (0.687‐0.859, M=0.788, SD=0.073), and F1-score (0.673‐0.793, M=0.745, SD=0.053). The moderate class, however, presented somewhat lower performance, with accuracy (0.761‐0.877, M=0.816, SD=0.055), sensitivity (0.676‐0.829, M=0.758, SD=0.071), specificity (0.788‐0.898, M=0.828, SD=0.047), precision (0.449‐0.546, M=0.485, SD=0.046), and F1-score (0.549‐0.646, M=0.588, SD=0*.041).*

For the PHQ-9, the rules showed balanced performance across severity levels. For the minimal depression class, the results were as follows: accuracy (range=0.803-0.927, M=0.859, SD=0.051), sensitivity (0.711-0.804, M=0.769, SD=0.044), specificity (0.916-0.981, M=0.939, SD=0.030), precision (0.878-0.935, M=0.899, SD=0.027), and F1-score (0.808-0.839, M=0.828, SD=0.014). Performance for the severe class was also balanced, with accuracy (0.862-0.889, M=0.879, SD=0.012), sensitivity (0.513-0.724, M=0.668, SD=0.104), specificity (0.945-0.958, M=0.948, SD=0.006), precision (0.598-0.864, M=0.752, SD=0.111), and F1-score (0.552-0.782, M=0.707, SD=0.105). The moderate/moderately severe class also showed reasonable performance, with accuracy (0.730-0.808, M=0.768, SD=0.031), sensitivity (0.749-0.892, M=0.803, SD=0.068), specificity (0.677-0.778, M=0.741, SD=0.048), precision (0.591-0.752, M=0.665, SD=0.066), and F1-score (0.688-0.816, M=0.726, SD= 0.059).

Overall, the PHQ-9 rules demonstrated more uniform performance across severity classes, whereas the GAD-7 rules performed strongly for minimal and severe cases but less so for moderate cases. The performance metrics obtained from the validation datasets were consistently aligned with those observed during model training (see Tables 2 and 3, as well as Supplementary Tables 2 and 3 in Multimedia Appendix 1).

## Discussion

### Principal Findings

This study investigated whether CART models could generate simplified yet accurate decision rules for stratifying anxiety and depression severity using GAD-7 and PHQ-9 items. The findings confirmed that a small number of items—2 for anxiety and 3 for depression—could produce high-performing rules, particularly for identifying minimal/mild and severe cases. Using bootstrapped resampling and cross-validation on a large nonclinical sample, we identified consistent and robust classification patterns, which were also reproduced in an independent dataset collected in Brazil with the same recruitment strategy, and also other four datasets in international contexts and different samples, providing evidence of external validity in relation to the target population. The resulting decision tree rules provided concise symptom pathways with strong performance across multiple metrics, supporting previous findings regarding simplified assessment methods [42]. The most frequent and best-performing rules involved only 2 or 3 items per scale, with no sociodemographic variable contributing to the classification. These findings underscore the potential of brief, rule-based models to enhance screening efficiency while maintaining classification accuracy [53,54].

These findings can be compared with those of other studies that used decision trees to analyze PHQ-9 and GAD-7 [43-46]. However, the models used in this study had the advantage of being less complex and using a large sample size. For example, in two studies that administered PHQ-9 to 2830 Japanese participants, the final model comprised all scale items and more than 20 rules [44,45], while this study had 4 rules. Another study that also applied PHQ-9 to 10,179 participants in Hong Kong found a final model with 8 items and more than 8 rules [43]. Furthermore, some studies with decision tree models included the total GAD-7 and PHQ-9 scores [46], with no straightforward severity stratification. To the best of our knowledge, this study combines the largest sample size with the simplest model. The findings suggest that abbreviated paths could optimize strategies for large-scale healthcare management [41].

Innovative approaches, such as logical rules for the GAD-7 and PHQ-9, as seen in this study, may serve as “shortcuts” for interpreting score combinations, enhancing the precision of large-scale screenings and targeted interventions. The approach leveraged ML models to identify adaptive conditional pathways within the full GAD-7 and PHQ-9 scales. This method preserved the comprehensiveness of the full symptom set while creating shortened, symptom-specific paths—similar to the GAD-2 and PHQ-2—but with the added ability to integrate other symptoms and severity levels in classifications. This approach balances respondent burden with a more nuanced and individualized evaluation of anxiety and depression symptoms, enhancing both screening precision and clinical utility [42]. By identifying the key items and logical rules, these results can support the development of scalable, cost-effective mental health screening models, addressing critical gaps in public health care, particularly in resource-limited settings.

The decision rules identified in this study offer a practical framework for rapid mental health screening, particularly in low-resource settings. By reducing the number of items needed for severity classification, the models allow nonspecialists to quickly identify minimal/mild, moderate, or severe cases. This can support timely decisions in primary care, schools, and community programs—settings where time and training are limited. The high performance for classifying extreme severity levels enables confident triage, guiding decisions from basic psychoeducation to referral for specialized care. These rules are also well-suited for integration into digital tools, expanding access to scalable and efficient assessment.

The current proposal offers significant advantages over GAD-2 and PHQ-2. First, the established ML rules enable the screening of symptom presence or absence and identification of severity levels, while providing detailed information on specific symptoms or sets of symptoms across the full scales. This approach moves beyond the fixed focus on 2 predetermined items in each scale. Second, through such rules, instruments as concise as the GAD-2 and PHQ-2 could be used while retaining the ability to detect other relevant symptoms, facilitating intervention design. The adaptiveness of these rules—the algorithms—allows for replication in diverse contexts, accommodating cultural and local specificities and facilitating the tracking of specific symptom trajectories across various scenarios. This adaptability is important as mental health expressions and symptomatology can vary significantly across cultural backgrounds (eg, [64-66]). Third, the rules can function as a preliminary screening step using if/else conditionals, reducing the length of the full scales, minimizing respondent burden, and lowering associated costs. Finally, it enables tailored interventions focused on specific symptoms and severity levels. For example, different strategies could be used for addressing PHQ-9 item 4 (fatigue) at minimal or mild versus moderate severity levels.

Our results demonstrated robustness when the procedure was repeated with different cutoff values for the scale scores, consistently identifying rules based on the same scale items. For example, in the PHQ-9 classifications, items 2 and 8 were most relevant for characterizing “severe” cases, whereas items 2 and 4 were most relevant for characterizing “minimal or mild” cases. However, a combination of these 3 items can show the path to the “moderate” levels: more related to the “minimal or mild” class while being closer to the “severe” level. GAD-7 findings could be interpreted similarly, where the moderate level shared some overlapping with “minimum or mild” and “severe” cases. Moreover, common limitations of decision trees, such as overfitting and instability, were not detected in this study [67]. Overfitting, often characterized by overly complex rules [54], was avoided as no rules contained more than 3 elements. Similarly, instability, which refers to changes in the tree structure due to variations in training data, was mitigated by identifying consistent patterns through resampling [68].

The decision tree rules demonstrated robust classification performance for the ”minimal or mild” and ”severe” categories; however, noticeably lower accuracy was observed for the ”moderate” severity class (GAD-7: 51%; PHQ-9: 67%). This finding suggests that the symptom profiles for moderate anxiety and depression may be inherently more nuanced and overlapping with those of minimal/mild and severe cases, leading to increased classification complexity. As illustrated by the prototypical decision trees, the “moderate” levels often required more complex rules and paths compared to the more distinct “minimal or mild” and “severe” levels, indicating a less clear-cut symptomatic boundary. This partial overlap with other severity levels points to an area for future methodological refinement. Future studies can improve the mid-severity level classification using alternative ML approaches. For instance, ensemble models (eg, random forests, gradient boosting machines), which combine multiple decision trees to reduce overfitting and enhance predictive accuracy, might be more adept at capturing the subtle distinctions within moderate symptom presentations. Additionally, exploring probabilistic thresholds rather than strict categorical cutoffs, or incorporating a fuzzy logic approach to severity classification, could provide a more nuanced and potentially more accurate assignment to the moderate range.

While the introduction highlighted the potential for decision trees to incorporate additional variables for adaptive and personalized analyses, the specific analyses revealed that sociodemographic factors (sex/gender, age, education level, and self-reported skin color/ethnicity) had minimal predictive value and did not emerge as part of any of the most frequently repeated classification rules for either the GAD-7 or PHQ-9. This suggests that within the context of our CART models and the specific severity stratification, the core symptom items were overwhelmingly more influential in classification. Consequently, detailed subgroup analyses based on these sociodemographic characteristics were not the most impactful predictive features for streamlined screening. Future research, perhaps using different ML approaches or focusing on distinct research questions, could more thoroughly investigate the interplay between sociodemographic factors and symptom presentation. By contrast, the lower impact of sociodemographic variables reinforces the principle that a broad approach to mental health literacy—including brief, self-guided interventions—can be directed at the general population with a reasonable margin of safety in reaching the intended audience. In other words, it offers a cost-effective strategy by enabling a concise and focused implementation to support scalable public policies.

These findings have significant practical implications for public mental health. The concise, rule-based models derived from the GAD-7 and PHQ-9 can be efficient “shortcuts” for large-scale mental health screening, particularly in resource-limited settings where extensive assessments are often impractical. For instance, in primary care settings, these simplified rules can empower health professionals, including nonspecialists, to rapidly identify disorders at varying risk levels (minimal/mild, moderate, and severe). This rapid stratification can facilitate tailored interventions, ranging from basic psychoeducation and self-guided resources for minimal/mild cases to immediate referral for more intensive support (eg, in-person appointments, specialized care) for moderate and severe cases. This directly supports the development of scalable and cost-effective mental health policies, enabling more efficient allocation of resources and improved access to care for a wider population.

While previous research has used similar methodologies to examine the PHQ-9 and GAD-7 scales using decision trees [43-46], our approach provides several key advantages, warranting a more critical comparison. For instance, some studies [44,45] are focused on individual PHQ-9 items; however, they often used different hyperparameters and, in some cases, dichotomized PHQ-9 scores, leading to substantially more complex decision trees with over 20 rules. By contrast, our study achieved highly accurate classifications with a significantly smaller set of rules (4 for PHQ-9), demonstrating a superior level of parsimony. Furthermore, some prior work [46] used total PHQ-9 and GAD-7 scores and incorporated sociodemographic variables directly into their models but, unlike our study, did not aim for severity stratification based on minimal item sets.

A critical advantage of our study lies in its large sample size, encompassing more than 20,000 participants from across all Brazilian states. This extensive and geographically diverse dataset significantly enhances the external validity and generalizability of our findings compared to those of previous studies with smaller cohorts. This large sample also allowed us to rigorously use bootstrapping and cross-validation, enabling robust estimation of rule performance and providing valuable dispersion measures that attest to the stability of the identified rules. While digital convenience sampling presents a limitation regarding full representativeness of the general population and potential selection bias toward individuals with digital access, the sheer scale and geographic breadth offer a substantial improvement in sample diversity compared to that in several previous studies in this domain. Thus, our work represents a significant advancement in optimizing mental health screening by combining the largest known sample size with the development of the most parsimonious and highly accurate decision tree models for GAD-7 and PHQ-9 severity classification.

Unlike fixed short scales such as the GAD-2 and PHQ-2, which rely on 2 specific symptoms and offer only binary outcomes, our models generate brief but adaptive rules that capture severity levels across a wider symptom range. While prior decision tree studies often retained most items and many rules, our models achieved high accuracy using only 2‐3 items and 4 rules per scale. These findings combine the simplicity of ultra-brief tools with the precision of full assessments, offering a more scalable and clinically informative alternative for public mental health applications.

The models identified in this study are concise and can be implemented in primary care by training health professionals in basic psychological care and developing specific intervention tools (eg, self-guided booklets, telephone consultations, and remote support) to manage “minimal or mild” symptoms. For moderate and severe cases, they serve as pathways for the rapid identification of individuals requiring more intensive support, such as in-person appointments, home visits, outpatient care, or even higher-level interventions such as inpatient treatment (eg, day hospitals or long-term hospitalization) [14,69,70]. These initiatives may be more feasible with straightforward instruments for evaluation and decision-making, improving the cost-benefit ratio and adding value to low-cost solutions for most demanding realities, such as mental health care in LMICs [8,40,71-75]. Thus, this study is aligned with the principles of a precision approach in the mental health field [76-78]—specifically, a precision health psychology approach.

### Limitations

This study has some limitations. First, the sample presented unbalanced sociodemographic characteristics, with more than 90% of participants identifying as female and an uneven distribution of skin color/ethnicity and education levels. Although variable importance analysis showed that sociodemographic factors—including sex/gender—contributed little to the final classification models (as none appeared in the most frequent rules), this representation pattern may limit the generalizability of the decision rules, particularly for male populations. Future studies should aim for a more balanced sample, especially with respect to sex/gender, which remains a persistent challenge across research contexts [79,80]. Although the CART models prioritized symptomatic items, it is possible that gender-specific symptom expressions or reporting styles may affect rule performance in different demographic contexts.

Second, although the dataset was large and included individuals from both rural and urban areas across Brazil, it is not representative of the general population. The recruitment was based on digital convenience sampling, using social media and electronic invitations, which may have introduced selection bias. Participants with limited internet access or low digital literacy may be underrepresented. Future studies should consider alternative recruitment methods to increase sample heterogeneity and representativeness.

Third, although robustness was supported by cross-validation, bootstrapping, and reproducibility in an independent national dataset, the reliance on digital convenience sampling may still limit representativeness. Future studies should extend external validation to different recruitment strategies and other nonclinical and clinical populations to strengthen generalizability. In addition, the choice of CART models was based on their interpretability and feasibility for large-scale use, but other machine learning techniques—such as ensemble models or deep learning—may offer alternative trade-offs between performance and complexity. Comparative studies could support future applications, especially in resource-limited settings.

### Conclusions

This study applied machine learning techniques—specifically decision trees combined with bootstrapping and cross-validation—to improve the classification of anxiety and depression severity based on GAD-7 and PHQ-9 items. Our findings show that just 2 items for anxiety and 3 for depression could generate highly accurate rules for identifying individuals with minimal or severe symptoms. Although the models performed well in distinguishing the extremes, their accuracy was lower for moderate cases, likely due to overlapping symptom patterns. These results suggest that caution is warranted when interpreting classifications in the middle severity ranges.

Simplified, rule-based models like those tested here can contribute to more efficient mental health care strategies. By using fewer items without compromising accuracy, they allow for faster assessments, better allocation of clinical resources, and quicker identification of cases requiring closer attention. In large-scale settings, this may support early interventions for mild cases and timely referrals for individuals showing more severe symptoms. Reducing the length and complexity of screening tools can also ease the burden of national data collection efforts, making it easier to integrate screening into population-based initiatives. Personalized guidance can then be offered based on specific symptom patterns—from psychoeducational materials and mental health literacy to behavioral interventions and clinical support. In this way, simple algorithms may support scalable and adaptive mental health surveillance strategies.

Further research should test these models in more diverse populations, using broader recruitment strategies beyond digital outreach to improve generalizability. While decision trees offer transparency and efficiency, comparative studies involving other machine learning methods—such as ensemble models or deep learning—could shed light on different cost-benefit trade-offs, especially in resource-limited settings. Longitudinal studies are also needed to evaluate the stability of these simplified models over time and their potential to monitor symptom progression or support personalized care. Finally, integrating these tools into digital health platforms or mobile applications could further enhance their accessibility and usefulness in real-world settings.

## Supplementary material

10.2196/72591 Multimedia Appendix 1 External validation of decision tree models for classifying anxiety and depression severity using GAD-7 and PHQ-9 scores: procedures and results.

## Abbreviations

AI: artificial intelligence
AUC: area under the curve
CART: classification and regression tree
CMDs: common mental disorders
CONEP: Conselho Nacional de Ética em Pesquisa
GAD-2: Generalized Anxiety Disorder Scale-2
GAD-7: Generalized Anxiety Disorder Scale-7
LMICs: low- and middle-income countries
ML: machine learning
OSF: Open Science Framework
PHQ-2: Patient Health Questionnaire-2
PHQ-9: Patient Health Questionnaire-9
ROC: receiver operating characteristic
