# Supplementary material for: Severity Classification of Anxiety and Depression Using Generalized Anxiety Disorder Scale and Patient Health Questionnaire: National Cross-Sectional Study Applying Classification and Regression Tree Models
Source: JMIR Public Health Surveill. 2025 Sep 30;11:e72591. doi: 10.2196/72591 (PMC12483340; doi:10.2196/72591)
Supplement: Multimedia Appendix 1 [file publichealth-v11-e72591-s001.pdf]

Supplementary Material for Severity Classification of Anxiety and Depression Using GAD-7 and PHQ-9: A National Cross-Sectional Study Applying CART Models

External validation was performed using four different datasets available in open repositories such as Figshare and Kaggle, along with a new dataset collected by the article's authors. We selected all available datasets on Figshare with item scores for GAD-7 and/or PHQ-9, and one large dataset from Kaggle. A summary of each dataset is presented in Table 1.

The most common rules obtained through decision trees were externally validated based on their accuracy, sensitivity (also known as recall), specificity, precision, and F1-score over the datasets previously mentioned (see Supplementary Figure 1 and Supplementary Tables 2 and 3). Precision indicates the model's reliability in predicting positive outcomes. Recall measures the proportion of true positive instances that were identified. The F1-score offers a single, balanced metric by calculating the harmonic mean of precision and recall. These metrics are particularly suitable for imbalanced datasets.

The metrics obtained showed that the rules for GAD-7 and PHQ-9 provided excellent identification of the minimal or mild strata, with high values of precision, recall, and F1-score. Their performance was also noteworthy for the severe strata of both GAD-7 and PHQ-9. For the severe strata of GAD-7, most precision values were higher than 0.800, and most recall and F1 values were higher than 0.700. For the severe strata of PHQ-9, most precision values were higher than 0.770, and most recall and F1 values were higher than 0.740. The strata with less noteworthy performance were moderate for GAD-7 and moderate and moderately severe for PHQ-9, which showed lower precision, recall, and F1 values.

Supplementary Table 1. Summary of Datasets Used for External Validation in a Cross-Sectional Study of Brazilian Adults (n = 20,585), 2024.

**Variables**		**Main Sample**	**VS 1 [1]**	**VS 2 [2]**	**VS 3 [3]**	**VS 4 [4]**	**VS 5 [5]**
Scales		GAD-7/PHQ-9	GAD-7/PHQ-9	GAD-7/PHQ-9	GAD-7	PHQ-9	GAD-7/PHQ-9
		F% (n = 20,585)	F% (n = 3,508)	F% (n=776)	F% (n=4,705)	F% (n=16,150)	F% (n=1,408)
Sex/ gender	Male	7.6 (1,564)	8.3 (290)	51.6 (400)	NF	73.3(10,882)	83.1(1,169)
	Female	91.5 (18,844)	90.8 (3,185)	48.4 (375)	NF	26.1(3,868)	16.9 (238)
	Non-binary	0.9 (177)	0.9 (33)	NF	NF	0.6 (90)	NF
Skin color/ ethnicity	White	50.5 (10,405)	49.7 (1,745)	NF	NF	NF	NF
	Black	10.5 (2,153)	10.9 (382)	NF	NF	NF	NF
	Parda (mixed race)	36.7 (7,575)	37.2 (1,304)	NF	NF	NF	NF
	Other	2.2 (452)	2.2 (79)	NF	NF	NF	NF
Education level	Up to high school	36.3 (7,062)	34.9 (1,223)	NF	NF	NF	NF
	Undergraduate	16.7 (3,440)	16.2 (568)	NF	NF	NF	NF
	Graduate	49.0 (10,083)	48.9 (1,717)	NF	NF	NF	NF
Age	18–29	18.1 (3,730)	18.7 (656)	99.6 (770)	2.4 (114)	42.3 (3475)	95.2 (1339)

Variables		Main Sample	VS 1 [1]	VS 2 [2]	VS 3 [3]	VS 4 [4]	VS 5 [5]
group (years)	30–39	28.8 (5,927)	28.4 (997)	0.4 (3)	6.7 (313)	40.9 (3355)	3.7 (52)
	40–49	29.5 (6,071)	27.9 (977)	NF	17.5 (822)	12.9 (1062)	0.6 (9)
	50–60	14.9 (3,062)	15.2 (533)	NF	29.2 (1372)	3.9 (318)	0.4 (6)
	More than 60	8.7 (1,795)	9.8 (345)	NF	44.2 (2079)	NF	NF

Notes. NF = data not found or unavailable.

- **[VS 1] Validation Sample 1.** Brazilian general population dataset including GAD-7 and PHQ-9 scores, with the same independent variables used in the main sample.
- **[VS 2] Validation Sample 2.** Mexican medical students. Both GAD-7 and PHQ-9 were applied. Available variables included sex/gender (binary only: male/female) and age groups from 18 to 39 years; no data were available on ethnicity or education level.
- **[VS 3] Validation Sample 3.** German cancer patients. Only the GAD-7 was applied. Data on age groups were available; no data were available on sex/gender, ethnicity, or education level.
- **[VS 4] Validation Sample 4.** Germany spoken general population sample. Only the PHQ-9 was applied. Data on sex/gender and age groups were available; no data were available on ethnicity or education level.
- **[VS 5] Validation Sample 5.** United Kingdom undergraduate students. Dataset includes both GAD-7 and PHQ-9. Data on sex/gender and age groups were available; no data were available on ethnicity or education level.

Supplementary Figure 1. Performance Metrics (Precision, Sensitivity, and F1-Score) of the Most Common Decision Tree Rules Applied to External Validation Datasets for the GAD-7 and PHQ-9 Scales in a Cross-Sectional Study of Brazilian Adults (n = 20,585), 2024.

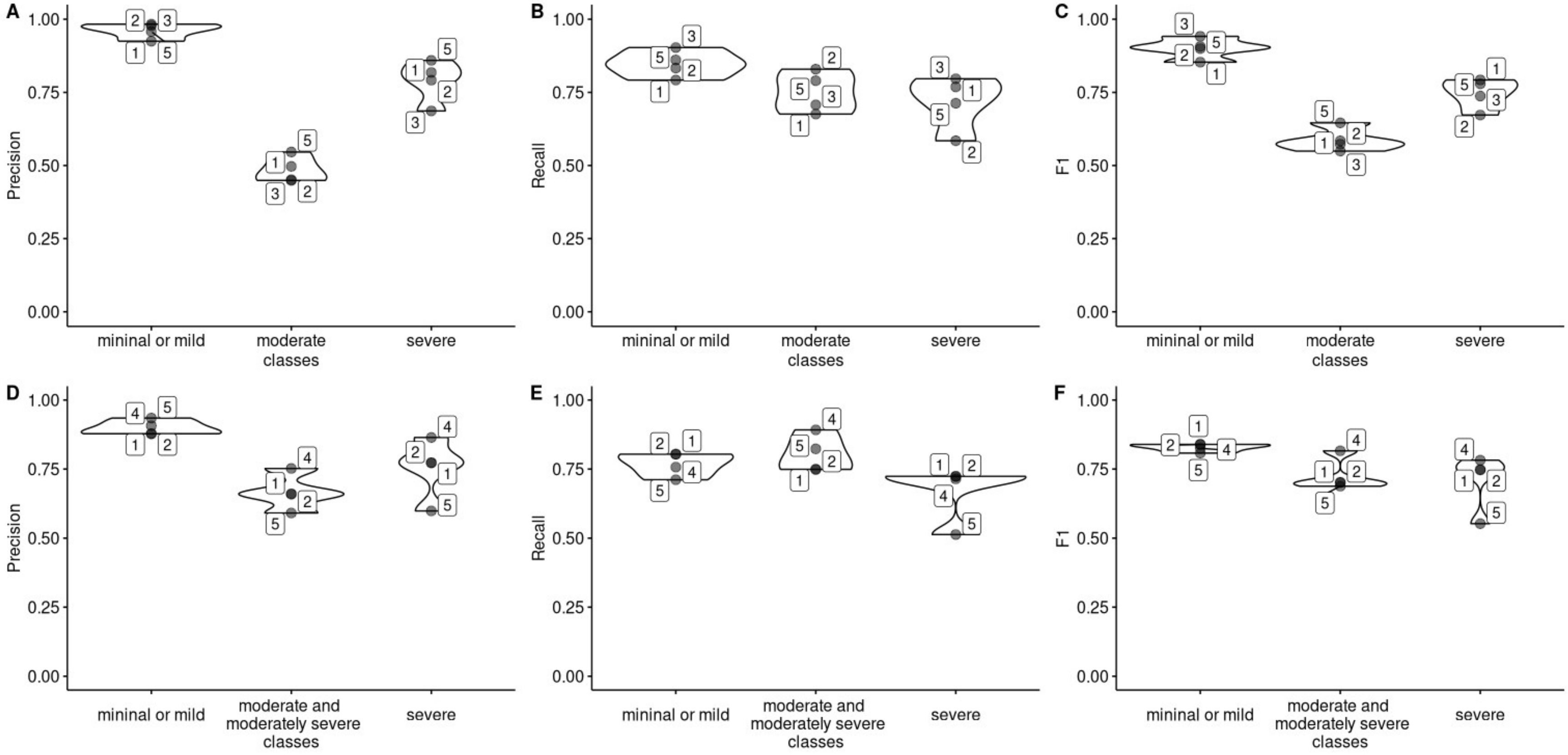

Supplementary Table 2. Performance Metrics of the Most Common Decision Tree Rules for the GAD-7 Scale Across External Validation Datasets in a Cross-Sectional Study of Brazilian Adults (n = 20,585), 2024.

Dataset	Class	Accuracy	Sensitivity	Specificity	Precision	F1-Score
1	Minimal or mild	0.855	0.791	0.942	0.924	0.853
	Moderate	0.760	0.675	0.787	0.496	0.572
	Severe	0.865	0.768	0.929	0.817	0.792
2	Minimal or mild	0.852	0.833	0.941	0.977	0.899
	Moderate	0.811	0.829	0.808	0.451	0.584
	Severe	0.944	0.584	0.985	0.791	0.672
3	Minimal or mild	0.902	0.903	0.915	0.983	0.941
	Moderate	0.877	0.707	0.897	0.449	0.549
	Severe	0.968	0.796	0.981	0.686	0.737
5	Minimal or mild	0.890	0.860	0.951	0.957	0.906
	Moderate	0.814	0.790	0.822	0.546	0.645
	Severe	0.899	0.712	0.966	0.859	0.779

Supplementary Table 3. Performance Metrics of the Most Common Decision Tree Rules for the PHQ-9 Scale Across External Validation Datasets in a Cross-Sectional Study of Brazilian Adults (n = 20,585), 2024.

Dataset	Class	Accuracy	Sensitivity	Specificity	Precision	F1-Score
1	Minimal or mild	0.853	0.803	0.916	0.878	0.839
	Moderate	0.767	0.749	0.778	0.659	0.701
	Severe	0.883	0.723	0.944	0.772	0.747
2	Minimal or mild	0.853	0.803	0.916	0.878	0.839
	Moderate	0.767	0.749	0.778	0.659	0.701
	Severe	0.883	0.723	0.944	0.772	0.747
4	Minimal or mild	0.927	0.757	0.980	0.906	0.825
	Moderate	0.807	0.891	0.730	0.752	0.816
	Severe	0.862	0.714	0.946	0.864	0.782
5	Minimal or mild	0.803	0.711	0.944	0.934	0.807
	Moderate	0.730	0.823	0.677	0.590	0.688
	Severe	0.889	0.512	0.957	0.598	0.552

Dataset References

[1] Grupo de Estudos e Pesquisas em Psicologia da Saúde (GEPPS/UFS). (2024). Basis for epidemiological surveillance in public mental health in Brazil (Year V, Dataset 2024, Time-point II). Health Psychology Laboratory, Federal University of Sergipe, Brazil. OSF. https://doi.org/10.17605/OSF.IO/NTSUF

[2] Mental health data of 776 Mexican medical students (PHQ-9, GAD-7 and Epworth Sleepiness Scale Scores). 2018. figshare. doi:10.6084/m9.figshare.1305817.v3.

[3] Screening for anxiety in patients with cancer: diagnostic accuracy of GAD-7 items considering lowered GAD-7 cut-offs. 2024. figshare. doi:10.6084/m9.figshare.27880509.v1.

[4] Burchert, Sebastian, André Kerber, Johannes Zimmermann, and Christine Knaevelsrud. 2019. “14-Day Smartphone Ambulatory Assessment of Depression Symptoms and Mood Dynamics in a General Population Sample: Comparison with the PHQ-9 Depression Screening.” Zenodo. https://zenodo.org/records/3384860.

[5] Comprehensive Data on the Prevalence of Psychiatric Symptoms in UK University Students: Data Files SPSS and XLSX Format and Variable Codes. 2023. figshare. doi:10.6084/m9.figshare.24052236.v2.
